# Topological properties and connectivity patterns in brain networks of patients with refractory epilepsy combined with intracranial electrical stimulation

**DOI:** 10.3389/fnins.2023.1282232

**Published:** 2023-11-23

**Authors:** Yulei Sun, Qi Shi, Min Ye, Ailiang Miao

**Affiliations:** ^1^Department of Neurology, Nanjing BenQ Medical Center, The Affiliated BenQ Hospital of Nanjing Medical University, Nanjing, Jiangsu, China; ^2^Department of Neurology, The Affiliated Brain Hospital of Nanjing Medical University, Nanjing, Jiangsu, China; ^3^Department of Neurology, The Affiliated Wuxi People’s Hospital of Nanjing Medical University, Wuxi, Jiangsu, China

**Keywords:** refractory epilepsy, functional magnetic resonance imaging, intracranial electrical stimulation, network connectivity patterns, small-world property

## Abstract

**Objective:**

Although intracranial electrical stimulation has emerged as a treatment option for various diseases, its impact on the properties of brain networks remains challenging due to its invasive nature. The combination of intracranial electrical stimulation and whole-brain functional magnetic resonance imaging (fMRI) in patients with refractory epilepsy (RE) makes it possible to study the network properties associated with electrical stimulation. Thus, our study aimed to investigate the brain network characteristics of RE patients with concurrent electrical stimulation and obtain possible clinical biomarkers.

**Methods:**

Our study used the GRETNA toolbox, a graph theoretical network analysis toolbox for imaging connectomics, to calculate and analyze the network topological attributes including global measures (small-world parameters and network efficiency) and nodal characteristics. The resting-state fMRI (rs-fMRI) and the fMRI concurrent electrical stimulation (es-fMRI) of RE patients were utilized to make group comparisons with healthy controls to identify the differences in network topology properties. Network properties comparisons before and after electrode implantation in the same patient were used to further analyze stimulus-related changes in network properties. Modular analysis was used to examine connectivity and distribution characteristics in the brain networks of all participants in study.

**Results:**

Compared to healthy controls, the rs-fMRI and the es-fMRI of RE patients exhibited impaired small-world property and reduced network efficiency. Nodal properties, such as nodal clustering coefficient (NCp), betweenness centrality (Bc), and degree centrality (Dc), exhibited differences between RE patients (including rs-fMRI and es-fMRI) and healthy controls. The network connectivity of RE patients (including rs-fMRI and es-fMRI) showed reduced intra-modular connections in subcortical areas and the occipital lobe, as well as decreased inter-modular connections between frontal and subcortical regions, and parieto-occipital regions compared to healthy controls. The brain networks of es-fMRI showed a relatively weaker small-world structure compared to rs-fMRI.

**Conclusion:**

The brain networks of RE patients exhibited a reduced small-world property, with a tendency toward random networks. The network connectivity patterns in RE patients exhibited reduced connections between cortical and subcortical regions and enhanced connections among parieto-occipital regions. Electrical stimulation can modulate brain network activity, leading to changes in network connectivity patterns and properties.

## 1 Introduction

Epilepsy is a neurological disorder characterized by recurrent and unprovoked seizures resulting from the intrinsic predisposition of the brain to generate unregulated electrical activity within its neural networks (Fisher et al., 2014). Although most epileptic patients show good responses to anti-seizure medications (ASMs), few patients continue to experience uncontrolled seizures despite receiving two ASMs (Schuele and Luders, 2008); this subgroup of epilepsy is called refractory epilepsy (RE) (Kwan et al., 2009). Frequent uncontrollable seizures can damage the developing cortical networks of the brain, leading to poor cognitive prognosis (Helmstaedter and Witt, 2017). It is essential to understand the pathogenesis underlying the electrical activity within brain networks to enable targeted interventions during the condition. Notably, functional imaging technology has been used frequently in neuropsychiatry (Li et al., 2017; Leitgeb et al., 2020; Wang et al., 2020; Song et al., 2021; Guan et al., 2022), thereby contributing significantly to the advancement of research on brain network disorders. Functional magnetic resonance imaging (fMRI), a non-invasive modality, can detect spontaneous neuronal activity within human brain networks at resting-state (Biswal et al., 1995). This technique has become increasingly essential for investigating healthy and dysfunctional brain function, facilitating a more comprehensive understanding of the mechanisms underlying seizure generation and propagation and the alterations within the framework of brain networks in epilepsy (Ogawa et al., 1992; Jiang et al., 2018).

Regarding brain networks, a brain region is defined as a node within the network. The direct topological connections or functional coupling correlations between these brain regions form the edges of the network (Guan et al., 2022). The rs-fMRI brain network approach has been used to identify significant topological characteristics in human brain functional networks through graph theory analysis; these features include small-world property and modular attributes (Wang et al., 2020). Furthermore, this method enables the investigation of functional connectivity between the entire brain and specific local brain regions (Guan et al., 2022). A brain network that exhibits the small-world property is referred to as a small-world network; this type of network is distinguished by a high degree of clustering coefficients (Cp) and a short average path length (Lp) (Watts and Strogatz, 1998; Bassett and Bullmore, 2017). It lies between random and regular networks, balancing the segregation and integration of information processing (Bassett and Bullmore, 2017). This organizational structure appears optimal for functioning in various complex systems, including brain networks (Bullmore and Sporns, 2009). Brain networks can be divided into modules, formed by a subset of highly connected nodes with limited connections to nodes in other modules (Meunier et al., 2010). These modules reflect the major functional systems of the brain, such as motor, somatosensory or visual functions (Stam and van Straaten, 2012). Frequently, damage to relevant modules or networks is associated with brain dysfunction in distinct neural networks.

A growing body of evidence suggests that these properties are altered in certain disease states; furthermore, these alterations in brain network connectivity can serve as valuable novel biomarkers for understanding the underlying psychopathology of diseases (Greicius, 2008; Liu et al., 2008; Zhang and Raichle, 2010; Vlooswijk et al., 2011; Tao et al., 2013; Sethi et al., 2016; Zheng et al., 2018; Tavakol et al., 2019; Drenthen et al., 2020; Tian et al., 2020; Song et al., 2021). For instance, Liu et al. (2008) discovered significant alterations in the small-world attribute of the prefrontal, parietal, and temporal lobes in patients with schizophrenia, compared to healthy controls. These variations also correlate with illness duration in schizophrenia (Liu et al., 2008). A study on Optic neuritis (Song et al., 2021) revealed that a decrease in Cp could signify reduced functional connectivity in specific brain regions due to severe demyelination, often seen in cases of axonal injury. Recent studies suggested that modular-related properties may be sensitive in reflecting brain changes in patients with major depressive disorder (Tao et al., 2013; Zheng et al., 2018; Tian et al., 2020). Studies on the topological properties of brain networks in epilepsy have also been reported. The study conducted by Drenthen et al. (2020) discovered a reduction in small-world organization in the brain networks of children with childhood absence epilepsy (CAE) in comparison to the controls. Additionally, the study revealed a positive correlation between Lp and disease duration as well as seizure frequency in CAE children. Studies of functional networks in temporal lobe epilepsy (TLE) have often reported increases in Lp (Vlooswijk et al., 2011; Sethi et al., 2016; Tavakol et al., 2019), and changes in these attributes have been associated with cognitive deficits (Vlooswijk et al., 2011).

These days, intracranial electrical stimulation has seen extensive use in treating psychiatric and neurological conditions, as well as in the preoperative localization of epilepsy (Lozano and Lipsman, 2013; Forouzannezhad et al., 2019; Thompson et al., 2020). Therefore, the impact of electrical stimulation on brain networks or brain functions, such as on perception (Parvizi et al., 2012), cognition (Parvizi et al., 2013), and emotion (Fried et al., 1998), has garnered considerable attention. However, studies on the properties of brain networks in relation to electrical stimulation are lacking due to its invasive nature. Patients with RE require surgery to intervene in the abnormal discharging of nerve cells in the brain due to uncontrollable seizures. This compensates for the fact that electrodes cannot be implanted in the brains of healthy people to study the effects of electrical stimulation. A novel approach integrates intracranial electrical stimulation with whole-brain neuroimaging in RE patients, enabling the quantification of acute long-range and network-level effects of the stimulation (Thompson et al., 2020). The method, which complements existing rs-fMRI network studies, can examine alterations in the functional connectivity of brain networks following electrode implantation, providing a more intuitive understanding of the impact of electrical stimulation on functional networks (Oya et al., 2017). Therefore, investigating the association between electrical stimulation and functional connectivity can provide insights into the precise impact of electrical stimulation on brain function; this includes its potential to facilitate or impede information transfer. Quantifiable indicators or evaluation criteria are needed to assess this stimulating effect. If there are network properties or phenotypes associated with electrical stimulation, this will provide useful information for the clinical treatment of RE.

Therefore, our study aimed to investigate the brain network characteristics of patients with RE concurrent electrical stimulation and obtain possible clinically significant biomarkers. To achieve this goal, the fMRI data of RE patients under combined electrical stimulation will undergo network connectivity analysis using graph theory topological properties. Firstly, we investigated the network properties in individuals with RE during resting-state and synchronous electrical stimulation in comparison to healthy controls to identify the main features of brain networks in RE patients. Then, we also compared the characteristics of brain networks before and after electrode implantation in the same patients, providing further evidence of the potential effects of electrical stimulation on network properties. This study is expected to provide more information for the diagnosis and intervention of RE.

## 2 Methods and materials

This study used the [Dataset] ds002799, available on the OpenNeuro data sharing platform.^1^ This dataset comprised 26 RE patients who underwent fMRI before and after electrode implantation. Most of them have no structural abnormalities or lesions in brain. The University of Iowa Institutional Review Board, Stanford University, and Caltech approved the study protocol. Informed consent was obtained from all the participants.

### 2.1 Data collection

All patients underwent anatomical MRI [T1-weighted (T1w) images] and rs-fMRI before electrode implantation. The T1w images were obtained using a 3 T GE Discovery 750w MRI equipped with a 32-channel head coil. The BRAVO sequence was used, with the following parameters: echo time (TE) of 3.376 ms, repetition time (TR) of 8.588 ms, flip angle of 12°, and voxel size of 1.0 × 1.0 × 0.8 mm. Participants were instructed to keep their eyes open during the rs-fMRI session before electrode implantation. Each session lasted 4.8 min and was conducted using a 32-channel head coil. The scan parameters were as follows: TR of 2,260 ms, TE of 30 ms, flip angle of 80°, and voxel size of 3.4 × 3.4 × 4.0 mm. Notably, no gap was present and the bandwidth was set at 2003 Hz/Px. The functional images after electrode implantation were scanned with simultaneous electrical stimulation on two distinct scanners before the surgery (Oya et al., 2017). The earlier images were obtained using a Siemens 3 T Trio scanner. In contrast, the later images were obtained using a 3 T Skyra (Siemens) due to specific collection conditions. The scan parameters used were as follows: TR of 3,000 ms; a delay of 100 ms was introduced in TR to coincide with the administration of electrical stimulation; TE of 30 ms; voxel size of 3 × 3 × 3 mm; flip angle of 90°; and bandwidth of 1934 Hz/Px. Notably, the authors of the raw data (Thompson et al., 2020) recommend treating the data obtained on the two scanners as comparable for group analyzes.

To minimize stimulation-induced MRI artifacts and the potential interactions between external electrical stimuli and radiofrequency or gradient switching-induced potentials in the electrodes, the delivery of electrical stimuli was interleaved with echo-planar imaging (EPI) volume acquisition, occurring within a 100 ms blank period devoid of scanner radiofrequency or gradient switching. Stimulation was blocked and organized in (approximately 30 s ON and OFF) with a total run duration of about 10 min for each electrical stimulation (es)-fMRI run of patients, with specific details varying slightly among patients. No significant difference was observed in head motion between no stimulation and stimulation epochs (Thompson et al., 2020). Most patients underwent multiple es-fMRI runs involving the implantation of several electrodes. These es-fMRI runs were included in the fMRI data preprocessing steps. The brain stimulation used bi-phasic charge-balanced square pulses characterized by a length of 50–90 ms, 8–12 mA, and 5–9 pulses administered at a stimulation rate of 100 Hz. The stimulation parameters, including amplitude, duration, and electrode position standard coordinates corresponding to the MNI152 template, can be found in the ieeg subdirectory of [Dataset] ds002799 on the OpenNeuro platform (Thompson et al., 2020). Table 1 presents the stimulation points for each patient. More details about the data collection can be found in these articles (Oya et al., 2017; Thompson et al., 2020).

**Table 1:** The RE patients’ demographics information.

Subject	Sex	Age (y)	Electrode stimulation sites/es-fMRI runs
292	F	50	Left posterior medial frontal (3runs) Left heschls gyrus (2runs)
303	F	34	Right amygdala (2runs)
307	M	30	Left heschls gyrus (3runs)
316	F	31	Right heschls gyrus (2runs) Right amygdala (1run) Right posterior hippocampus (1run)
320	F	50	Right frontal lobe (1run) Left amygdala (2runs) Right heschls gyrus (1run)
330	M	43	right parietal lobe (1run) Right amygdala (1run) Right amygdala and planum temporal simultaneously (1run) Right superior posterior occipital lobe (1run)
331	M	35	Left amygdala (1run) Right frontal lobe (1run)
334	M	39	Right planum temporal (3runs) Left amygdala (4runs) Right posterior hippocampus (1run) Left and right amygdala (1run)
335	M	31	Right heschls gyrus (1run) Left anterior insula (1run)
352	M	31	Left heschls gyrus (1run)
357	M	36	Left posterior medial frontal lobe (1run)
372	M	34	Left heschls gyrus (1run)
395	M	13	Left amygdala (2runs)
399	F	22	Right anterior cingulate (2runs) Right amygdala (1run)
400	M	59	Left heschls gyrus (1run)
405	M	19	Right amygdala (1run) Left anterior insula orbitofrontal cortex (1run) Right hippocampus (1run)
413	M	22	Right frontal operculum ofc (1run) Right cingulate (1run) Right inferior posterior insula (1run)

The Table 1 documents the basic information of RE patients included in the network connectivity analysis, including age, sex, the brain regions of electrode stimulation and electrode stimulation corresponding to es-fMRI runs. RE, refractory epilepsy; y, years; F, female; M, male; es-fMRI, the fMRI concurrent electrical stimulation of RE patients.

For comparative analysis of network connectivity with healthy controls, we used the [Dataset] Berlin_Margulies from the 1,000 Functional Connectomes Project.^2^ This dataset comprised structural and functional images of 26 healthy controls (without neurological or psychiatric disorders) aged 23–44 years. All imaging scans were conducted using a Siemens 3 T Trio scanner. The T1w imaging parameters were as follows: voxel size of 1 × 1 × 1 mm, TR of 2.3 s, TE of 2.98 ms, inversion time (TI) of 900 ms, flip angle of 9°, and bandwidth of 240 Hz/Px. Regarding the fMRI data acquisition, the following parameters were used: 34 slices, voxel size of 3 × 3 × 4 mm, TR of 2.3 s, TE of 30 ms, flip angle of 90°, a total of 200 measurements were acquired in 7.45 min, no delay in TR, and a bandwidth of 2,232 Hz/Px.

The functional scans obtained before electrode implantation are resting-state BOLD fMRI data and were referred to as “rs-fMRI” in our study. The functional data obtained after electrode implantation, scanning with simultaneous electrical stimulation, was denoted as “es-fMRI” in our study. As no task was performed during the es-fMRI scans, and the electrical stimulation did not affect perception or behavior noticeably, the es-fMRI scans can be treated as “resting-state” fMRI (Thompson et al., 2020). When comparing patients with RE and healthy controls, the fMRI data collected from the healthy controls group was labeled as “HC-fMRI.” High-quality T1w images were acquired before any neurosurgical intervention, as the original data researchers recommended and supported by experimental evidence (Thompson et al., 2020). Therefore, during fMRI data pre-processing and analysis, T1w images were used for registration with the images of rs-fMRI and es-fMRI. Consistent with the findings of the previous study (Thompson et al., 2020), we successfully registered es-fMRI images with the T1w images before electrode implantation.

### 2.2 fMRI data pre-processing

The fMRI data from all enrolled participants were pre-processed using the GRETNA toolbox^3^ (Wang et al., 2015) in the MATLAB 2013b platform (Mathworks, Natick, MA, United States). The pre-processing protocols involved several steps, including the removal of the initial 10 volumes, correction of slice timing, correction of head motion, normalization of spatial data through T1 segmentation, elimination of linear trends, temporal band filtering (0.01–0.08 Hz), and regression of nuisance signals, including 24-parameter head motion profiles and cerebrospinal fluid and white matter signals. Participants exhibiting excessive head movement (translation >3.0 mm or rotation >3.0° in any direction) and those with framewise displacement (FD) > 0.5 mm were excluded. Upon completion of fMRI data pre-processing, 18 RE patients and 23 healthy controls were included.

### 2.3 Network connectivity construction and analysis

**Figure 1:**
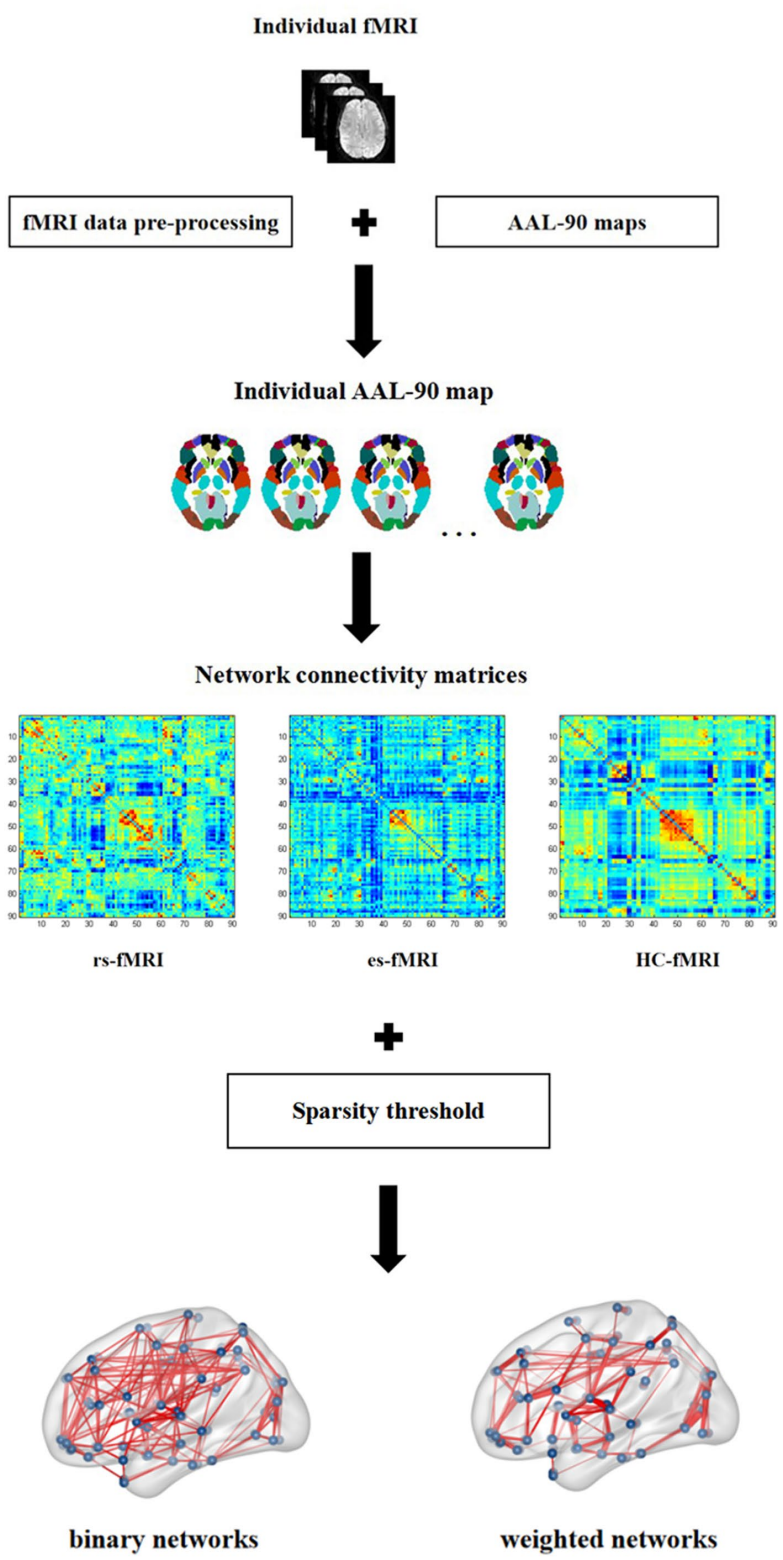
Network connectivity construction. In this figure, individual fMRI represents the fMRI image of each subject enrolled in present study, which is pre-processed on the standard AAL-90 atlas to construct the functional connectivity matrix for each subject. The three connectivity matrices in the figure show the example of one subject, respectively, selected from rs-fMRI, es-fMRI and HC-fMRI. Finally, the binary networks and weighted networks are constructed using sparsity threshold method. rs-fMRI, the resting-state fMRI of RE patients; es-fMRI, the fMRI concurrent electrical stimulation of RE patients; HC-fMRI, healthy controls’ fMRI.

This study analyzed the differences in network connectivity between RE patients (including rs-fMRI and es-fMRI) and healthy controls. Furthermore, it assessed the effects of electrical stimulation on fMRI network connectivity. The AAL-90 atlas was used to construct 90 × 90 network connectivity matrices for each participant. The parcellation atlas provides essential information about the order, location, and names of each node stored in the toolbox (… \GRETNA\Templates). The resulting network connectivity matrices from various participants were then converted into two types of networks: binary and weighted. This conversion was achieved using sparsity thresholding techniques. These networks were used in our study to describe the characteristics of functional network connectivity. A recent study has demonstrated detailed algorithms for generating binary and weighted networks (Wang et al., 2015). The difference between binary and weighted networks primarily depends on whether connectivity strength is considered (Wang et al., 2015). Figure 1 shows the process for constructing network connectivity. The concept of sparsity, defined as the ratio of actual connections (E) to the total number of potential connections [N(N-1)/2] (Zalesky et al., 2012), has received significant attention in the scientific literatures. It has been used to investigate small-world organization across various connection densities. Previous studies have examined densities as low as 1–5% (Achard et al., 2006; Kitzbichler et al., 2011) and as high as 50% (Lynall et al., 2010). To ascertain small-world organization, the minimum connection density is often determined by ensuring that k > log(N), where k represents the mean node degree (Achard et al., 2006). Applying this principle to a network of dimensions *N* = 90 results in a minimum connection density of 5% (Zalesky et al., 2012). To ensure meaningful network connectivity and facilitate the estimation of small-world organization, the sparsity threshold used in our study was set at 5–35%, with increments of 1%. For our network connectivity analysis, we used the rs-fMRI data and 50 runs of es-fMRI data from 17 RE patients and fMRI data from 22 healthy controls.

Our study analyzed various topological properties in brain networks, including global and nodal parameters. The global network properties included characteristics of small-world property and network efficiency, such as global network efficiency (Eg) and local network efficiency (Eloc). The small-world property was evaluated through these parameters, such as the Cp, Lp, normalized clustering coefficient (γ), normalized characteristic path length (λ), and small-worldness (σ). Notably, γ was calculated as the ratio of Cp to Cprand, whereas λ was computed as the ratio of Lp to Lprand. These reference values, Cprand and Lprand, were derived from randomized networks generated by using a Markov-chain algorithm (Maslov and Sneppen, 2002; Sporns and Zwi, 2004) within the GRETNA toolbox; this maintains an equivalent number of nodes and edges, as well as comparable degree distribution to the actual brain networks (Wang et al., 2015). Small-world networks exhibit a significantly elevated mean Cp akin to regular lattice networks (γ > 1), along with small Lp comparable to random networks (λ ≈ 1) (Watts and Strogatz, 1998). Cp, defined as the proportion of connections established between a node’s neighbors, provides insight into the extent of connections within a local cluster (Leitgeb et al., 2020). A pronounced Cp signifies efficient local information transfer and resilience against random attacks and subsequent node failures (Bullmore and Sporns, 2009; Rubinov and Sporns, 2010). In contrast, the Lp refers to the minimum number of edges necessary for traversing from one node to another (Van Straaten and Stam, 2013). The observed Lp within brain networks further emphasizes efficient parallel information transfer and effective global integration (Van Straaten and Stam, 2013).

Our study has also computed various nodal properties concerning network connectivity, including nodal clustering coefficient (NCp), nodal characteristic shortest path length (NLp), betweenness centrality (Bc), and degree centrality (Dc). Bc quantifies the impact of a node on the information flow in the graph, whereas Dc measures the number of direct connections a given node maintains with other nodes in the graph (Guan et al., 2022). To evaluate the global and nodal topological characteristics of the brain networks, the area under the curve (AUC) was computed for each parameter. This metric, calculated independently at the single threshold, is highly sensitive to the abnormal topological structure of brain diseases (Zhang et al., 2011). In the group analysis, AUC values of global and nodal network properties are used to compare the differences in network connectivity between rs-fMRI, es-fMRI and HC-fMRI.

To compare the distribution characteristics of fMRI network connectivity between RE patients (including rs-fMRI and es-fMRI) and healthy controls, network connectivity modules were constructed using a structural division of 90 regions of interest in AAL-90 atlas. These regions of interest were categorized into six sub-modules: frontal lobe, prefrontal lobe, subcortical areas, temporal lobe, occipital lobe, and parietal lobe. Our study computed the connectivity strength of intra-and inter-modules among rs-fMRI, es-fMRI, and HC-fMRI. In total, the connectivity strength from 15 inter-modular connections and 6 intra-modular connections were used to analyze modular connectivity patterns.

A comparative observation was performed using the fMRI data of same patients to conduct a more comprehensive analysis of brain networks pre-and post-electrode implantation. Multiple runs of es-fMRI images from the same patient were compared with their rs-fMRI images, using small-world parameters and network efficiency as comparable quantitative measures. These network properties values of es-fMRI runs were divided into two categories with the values greater than and less than rs-fMRI, and the differences between them were assessed to determine whether the parameters distribution originated from the same population.

All stages of image pre-processing, network construction, and analyses were performed using the GRETNA toolbox. The results about nodal properties and the network connectivity patterns were visualized using the BrainNet Viewer toolbox^4^ (Xia et al., 2013).

### 2.4 Statistics

The connectivity topological properties, including global, nodal, and modular parameters, in brain networks of rs-fMRI, es-fMRI, and HC-fMRI were compared using a one-way analysis of variance (ANOVA) test; the analysis controlled for the covariates of mean FD and age of each participant. Non-parametric tests were used when data did not meet the criteria for normal distribution. *p*-values <0.05 were considered statistically significant. *Post hoc* tests were performed between any two groups in cases where the ANOVA test revealed significant differences. Bonferroni correction procedure was used to evaluate the multiple comparisons of nodal parameters and modular analysis. Fisher’s exact test was used to determine the consistency of small-world parameters and network efficiency between es-fMRI runs and rs-fMRI data for individual comparisons. Statistical analyses were performed using SPSS version 20 (SPSS, Inc., Chicago, IL, United States).

## 3 Results

The network analysis finally included 17 patients with RE and 22 healthy controls in our study. No significant differences were observed between both groups with respect to age (*p* = 0.171) and sex (*p* = 0.325). The RE patients had an average age of 34.06 ± 11.85, among whom 5 were females and 12 were males. The healthy controls had an average age of 29.73 ± 4.86, including 12 females and 11 males. The demographics of all study participants are presented in Tables 1, 2.

**Table 2:** The healthy controls’ demographics information.

Subject	Sex	Age (y)
06204	M	34
12855	M	33
18913	F	29
23506	F	27
27536	M	25
27711	F	26
27797	M	31
28092	F	26
33248	F	28
38279	M	29
47066	F	26
47791	M	31
49134	M	44
54976	M	37
57028	F	37
67166	F	32
75506	M	28
85681	F	26
86111	F	24
91966	F	27
95068	M	26
97162	M	28

The Table documents basic information of healthy controls included in the network connectivity analysis, including age and sex. y, years; F, female; M, male.

### 3.1 Global network properties

**Figure 2:**
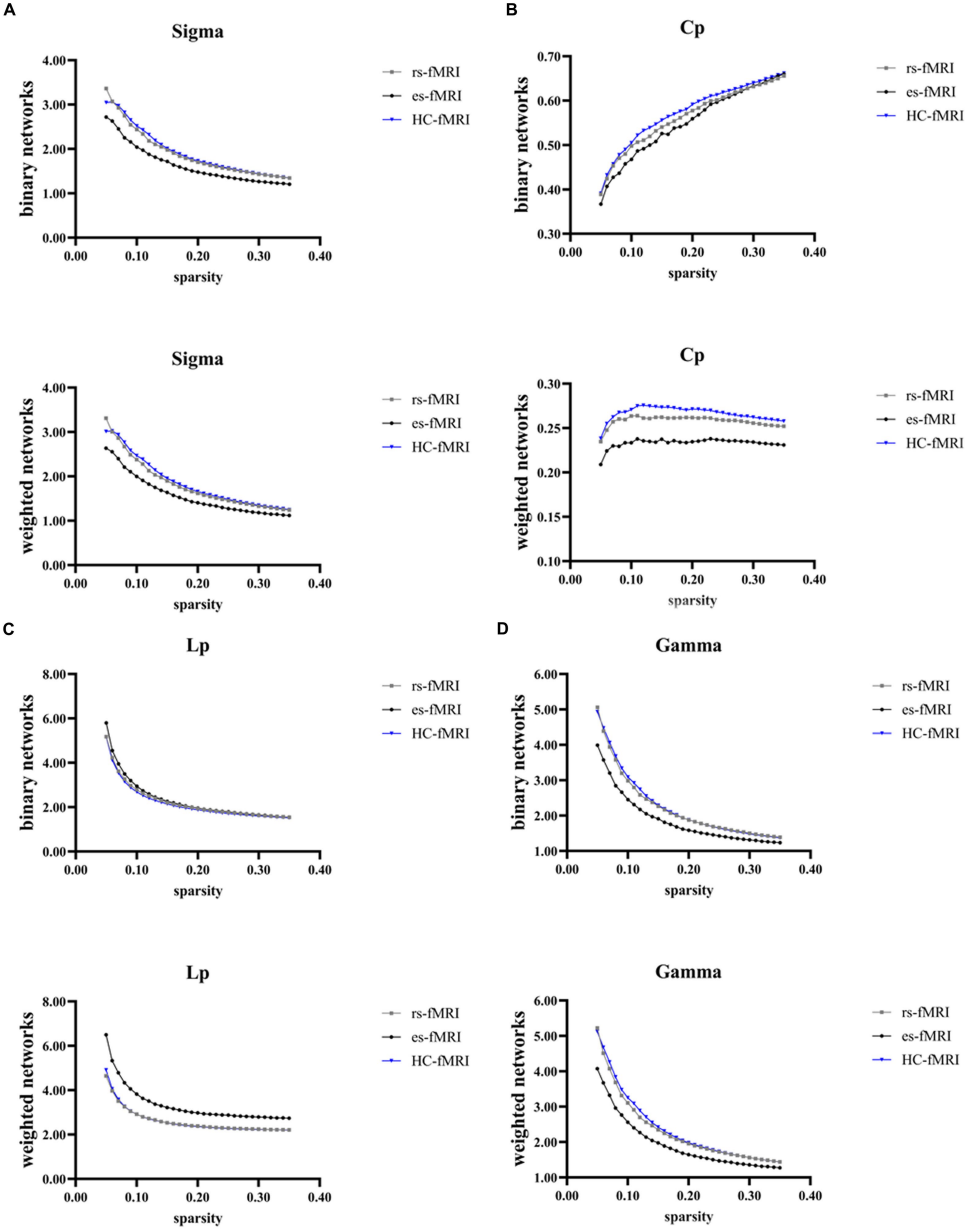

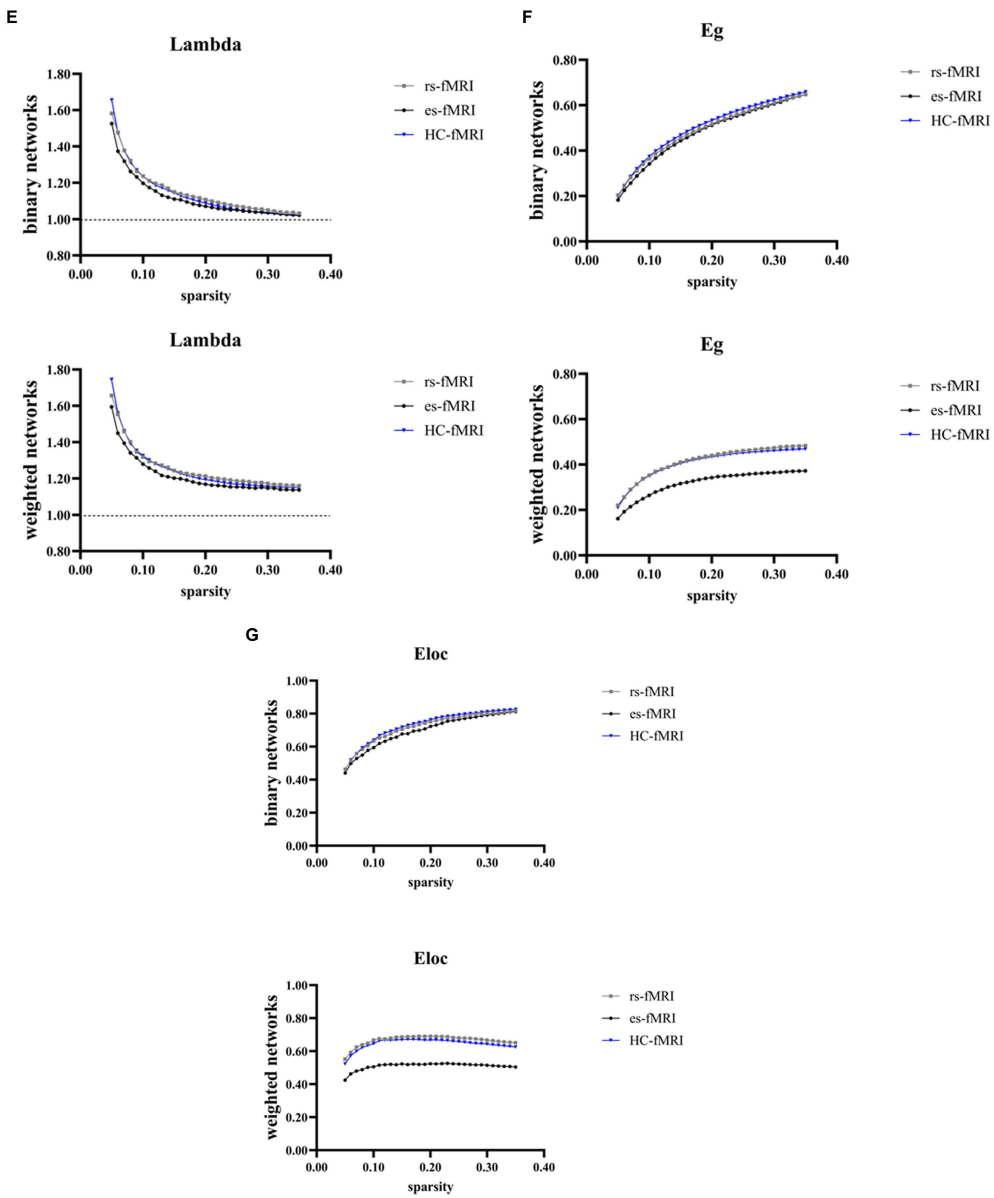
Global network properties varying with changes in sparsity. The figure illustrates the global network property parameters at different sparsity thresholds. The X-axis denotes the sparsity threshold, while the Y-axis represents the corresponding property values. The observed alterations in the line patterns indicate a consistent change in the network properties among the rs-fMRI, es-fMRI and HC-fMRI with varying sparsity levels. **(A–G)** The figures illustrate the variations in Sigma, Cp, Lp, Gamma, Lambda, Eg and Eloc values in response to changes in sparsity. Sigma (σ), small-worldness; Cp, clustering coefficient; Lp, characteristic path length; Gamma (γ), normalized clustering coefficient; Lambda (λ), normalized characteristic path length; Eg, global network efficiency; Eloc, local network efficiency; rs-fMRI, the resting-state fMRI of RE patients; es-fMRI, the fMRI concurrent electrical stimulation of RE patients; HC-fMRI, healthy controls’ fMRI.

**Figure 3:**
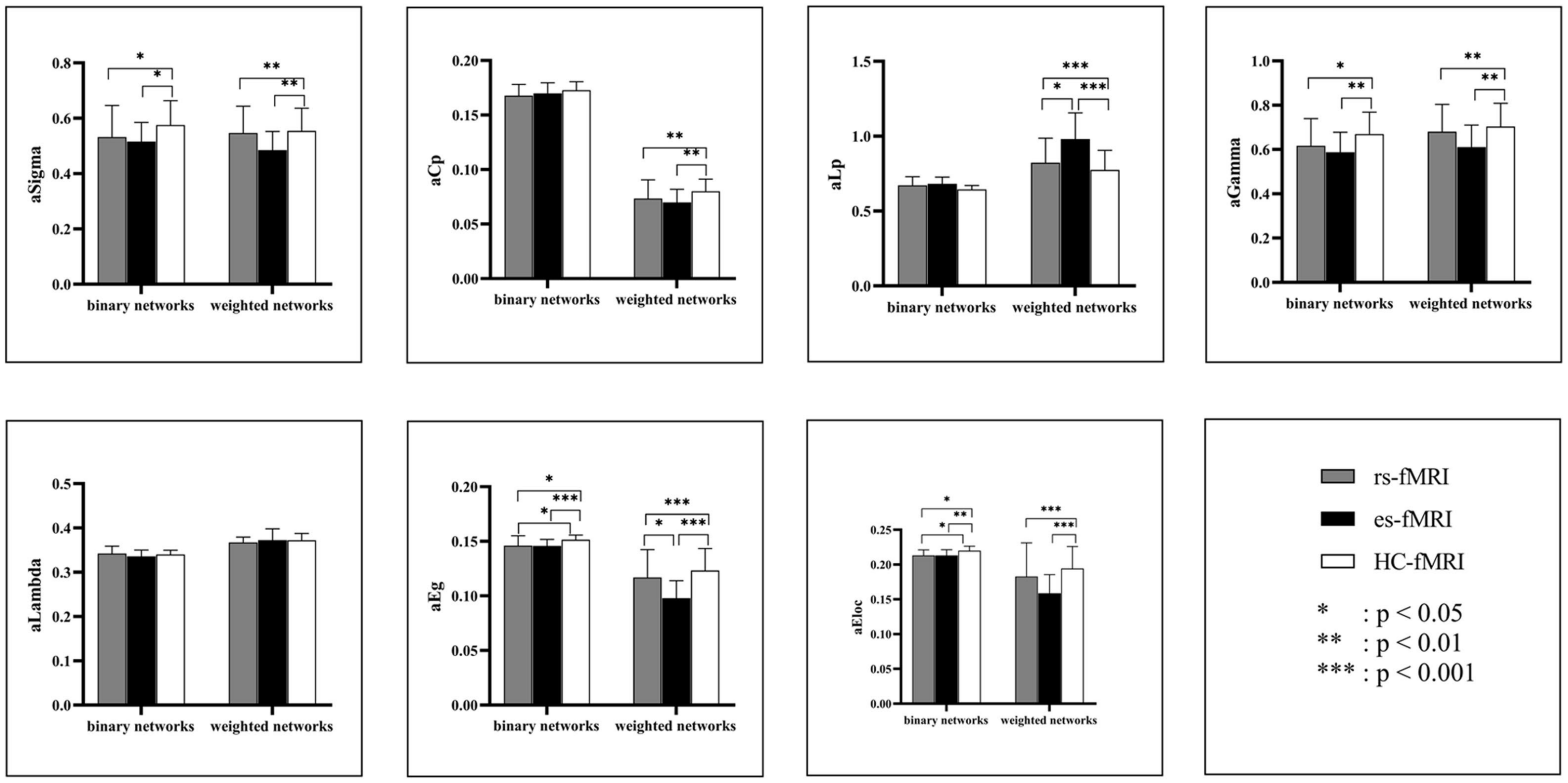
Comparisons of global network properties. The figure shows the AUC values comparison of global network properties of rs-fMRI, es-fMRI, and HC-fMRI in both binary and weighted networks under all sparsity thresholds. Network properties with statistical differences after multiple correlations are marked with black asterisks. The “a” refers to the AUC values of these network properties. AUC, area under the curve; Sigma (σ), small-worldness; Cp, clustering coefficient; Lp, characteristic path length; Gamma (γ), normalized clustering coefficient; Lambda (λ), normalized characteristic path length; Eg, global network efficiency; Eloc, local network efficiency; rs-fMRI, the resting-state fMRI of RE patients; es-fMRI, the fMRI concurrent electrical stimulation of RE patients; HC-fMRI, healthy controls’ fMRI.

The results revealed a consistent trend of change in network topological properties among RE patients and healthy controls as the sparsity threshold varied (Figure 2). As the network’s sparsity threshold increased, σ, Lp, γ, and λ demonstrated a decrease in both binary and weighted networks. Conversely, Cp and network efficiency (Eg and Eloc) increased with varying sparsity thresholds in both network types. Upon comparing the global property parameters across three groups (rs-fMRI, es-fMRI and HC-fMRI), significant differences were observed in σ, γ, Eg, and Eloc among the three groups in both types of networks (Figure 3). Notably, significant differences were observed in Cp and Lp among the three groups in weighted networks (Figure 3). However, in binary networks, no significant differences were observed in Cp (*p* = 0.174) and Lp (*p* = 0.101) among the three groups. The binary networks of es-fMRI showed slightly elevated Cp values compared to those of rs-fMRI, although this difference was not statistically significant. The brain networks derived from the es-fMRI exhibited lower σ, γ, Eg, and Eloc and higher Lp values than those from the rs-fMRI and HC-fMRI (Figure 3). Although no statistically significant differences were observed in small-world parameters between the rs-fMRI and HC-fMRI, the rs-fMRI group demonstrated lower σ, Cp, γ and higher Lp values. The binary networks exhibited significant differences in Eg and Eloc between the rs-fMRI and HC-fMRI (Figure 3). The λ demonstrated no significant differences among the three groups, regardless of whether binary (*p* = 0.429) or weighted networks (*p* = 0.578) were considered.

### 3.2 Nodal network properties

**Figure 4:**
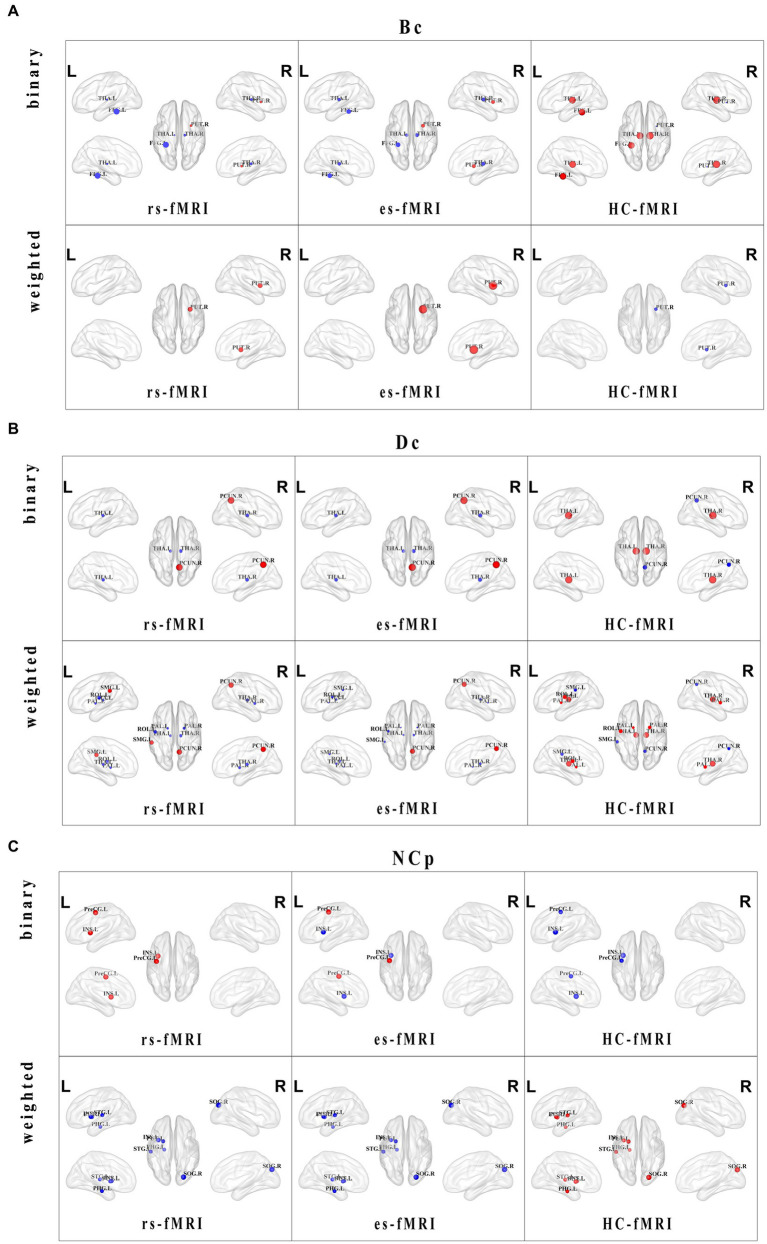
Distributions and comparisons of nodal network properties. The figure illustrates the distribution of nodal network properties within the brain, highlighting significant differences between RE patients (including rs-fMRI and es-fMRI) and healthy controls in both binary and weighted networks. Specifically, the figures respectively display the comparisons of Bc **(A)**, Dc **(B)** and NCp **(C)** in the two types of networks across three groups of individuals. Nodes with distinct differences are exhibited in figures, and marked with their brain region abbreviations. The varying sizes of nodes reflect the different values of the nodal properties, where the red and blue dots, respectively, indicate the increased and decreased nodal property values in brain regions. Bc, betweenness centrality; Dc, degree centrality; NCp, nodal clustering coefficient; FFG.L, left fusiform gyrus; PUT.L, left putamen; PUT.R, right putamen; THA.L, left thalamus; THA.R, right thalamus; PAL.L, left pallidum; PAL.R, right pallidum; PCUN.R, right precuneus; ROL.L, left rolandic operculum; SMG.L, left supramarginal gyrus; SOG.R, right superior occipital gyrus; INS.L, left insula; PHG.L, left parahippocampal gyrus; PreCG.L, left precental gyrus; STG.L, left superior temporal gyrus.

Regarding nodal properties, Bc, Dc, and NCp exhibited differences among the three groups (rs-fMRI, es-fMRI and HC-fMRI) in binary and weighted networks (Figure 4). However, NLp displayed no significant differences among the three groups in either network type. In binary networks, specific nodes, such as the left fusiform gyrus (FFG.L), right putamen (PUT.R), left thalamus (THA.L), and right thalamus (THA.R), demonstrated distinct Bc values (Figure 4A). Similarly, in weighted networks, the Bc of PUT.R exhibited differences. Notably, healthy controls exhibited higher Bc values in the bilateral thalamus and FFG.L compared to RE patients (including rs-fMRI and es-fMRI) in binary networks. Conversely, the rs-fMRI and the es-fMRI of RE patients exhibited higher Bc values in PUT.R compared to healthy controls in both types of networks. The Dc of the right precuneus (PCUN.R), THA.L, and THA.R exhibited significant differences among the three groups in binary networks (Figure 4B), whereas the Dc of left rolandic operculum (ROL.L), left supramarginal gyrus (SMG.L), PCUN.R, left pallidum (PAL.L), right pallidum (PAL.R), THA.L and THA.R exhibited differences in weighted networks. Notably, the rs-fMRI and the es-fMRI of RE patients exhibited increased Dc values in PCUN.R compared to healthy controls in binary and weighted networks. Furthermore, the rs-fMRI exhibited higher Dc values in SMG.L compared to HC-fMRI and es-fMRI in weighted networks. Conversely, in binary networks, THA.L and THA.R demonstrated higher Dc values in healthy controls than RE patients (including rs-fMRI and es-fMRI). Moreover, in weighted networks, ROL.L, PAL.L, PAL.R, THA.L, and THA.R exhibited higher Dc values in healthy controls than RE patients (including rs-fMRI and es-fMRI). The left precentral gyrus (PreCG.L) exhibited higher NCp value in RE patients (including rs-fMRI and es-fMRI) compared to healthy controls in binary networks. By contrast, healthy controls demonstrated increased NCp in left parahippocampal gyrus (PHG.L), right superior occipital gyrus (SOG.R), left putamen (PUT.L), and left superior temporal gyrus (STG.L) compared to RE patients (including rs-fMRI and es-fMRI) in weighted networks (Figure 4C). The binary networks of rs-fMRI exhibited increased NCp in left insula (INS.L) compared to HC-fMRI, while the INS.L of es-fMRI showed the lowest NCp values in two types of networks.

### 3.3 Network connectivity patterns

**Figure 5:**
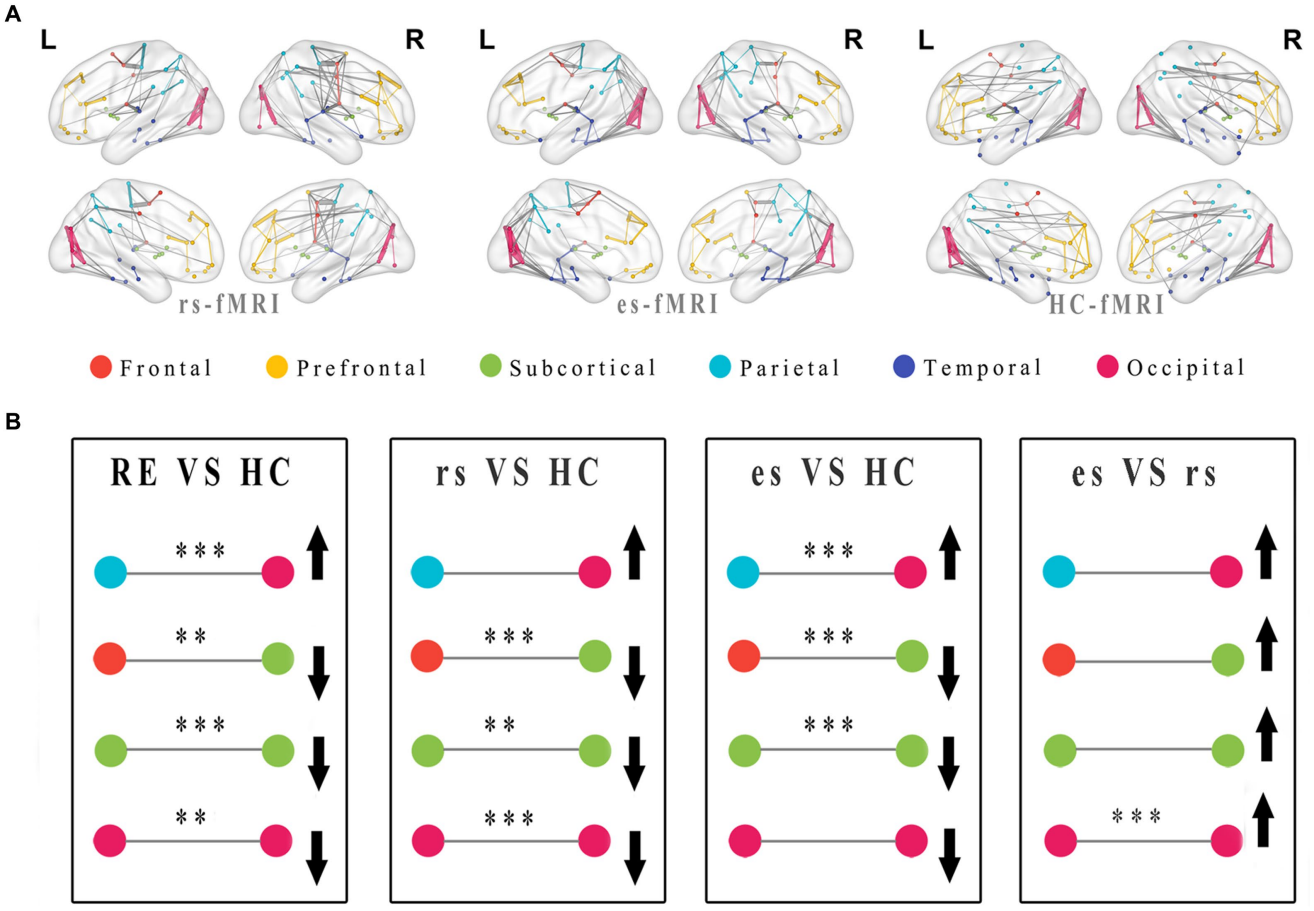
Modular distributions and comparisons of network connectivity patterns. The group average functional connectivity matrix of rs-fMRI, es-fMRI and HC-fMRI was used to construct group average connectivity map with modular division, and the connections greater than the sparsity threshold value of 0.05 were shown in this figure **(A)**. In this figure, the internal connections of the modules are connected with lines of the same color as the modules, and the connections between different modules are connected with gray lines. The thickness of the line indicates the connectivity strength of the connections between brain regions. **(B)** The figure shows the modularization analysis of RE patients and healthy controls. The black asterisks were used to exhibited significant differences after multiple comparisons, and the black arrows were utilized to indicate relative increase or decrease in module connectivity strength. Where the * means *p* < 0.05, ** means *p* < 0.01, *** means *p* < 0.001. RE, refractory epilepsy patients; HC, healthy controls; rs, the resting-state fMRI of RE patients; es, the fMRI concurrent electrical stimulation of RE patients.

The network connectivity distributions among brain modules of three groups (rs-fMRI, es-fMRi and HC-fMRI) were displayed in Figure 5A. The modular analysis revealed significant differences in inter-regional connectivity, such as the connections between frontal lobe module and subcortical regions, and the connections between parietal lobe module and occipital lobe module (Figure 5B). Internal connectivity differences were observed within subcortical areas and the occipital lobe module (Figure 5B). Compared to healthy controls, RE patients demonstrated reduced connectivity between the frontal and subcortical areas while exhibiting enhanced connectivity between the parietal and occipital areas. Notably, modular connectivity strength within the subcortical and occipital networks in RE patients was lower compared to healthy controls. In all modular differences, es-fMRI brain networks exhibited increased connectivity strength compared to those from rs-fMRI.

### 3.4 Individual network connectivity comparison between rs-fMRI and es-fMRI

**Figure 6:**
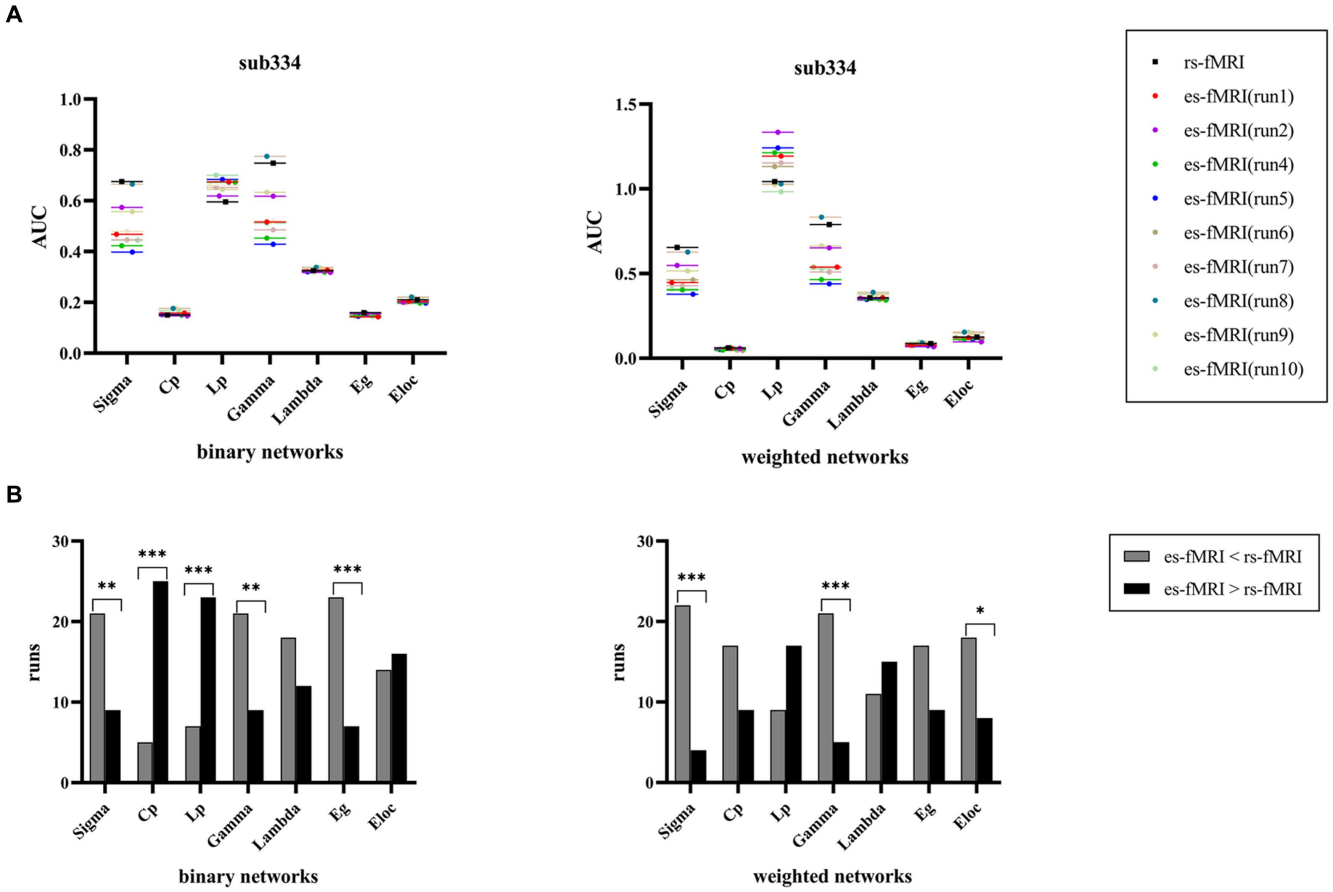
Comparisons of individual network properties between rs-fMRI and es-fMRI. **(A)** The figure shows the distribution of AUC values of global network properties between rs-fMRI and es-fMRI in two types of networks using sub334 as an example. **(B)** The figure shows the comparison of network connectivity properties between rs-fMRI and es-fMRI, and black asterisks mark significant differences in brain networks between the two states. AUC, area under the curve; Sigma (σ), small-worldness; Cp, clustering coefficient; Lp, characteristic path length; Gamma (γ), normalized clustering coefficient; Lambda (λ), normalized characteristic path length; Eg, global network efficiency; Eloc, local network efficiency; rs-fMRI, the resting-state fMRI of RE patients; es-fMRI, the fMRI concurrent electrical stimulation of RE patients.

The findings of sub334 were used to exemplify the differences in network connectivity between rs-fMRI and es-fMRI (Figure 6A). Notably, properties, such as σ, Cp, Lp, γ, Eg, and Eloc, exhibited differences, whereas λ showed no significant difference between rs-fMRI and es-fMRI. The rs-fMRI brain networks exhibited elevated σ and γ values, whereas the es-fMRI brain networks demonstrated relatively increased Lp values (Figure 6B) in both network types. Additionally, Eg in binary networks and Eloc in weighted networks of rs-fMRI were higher than those of es-fMRI. The Cp of es-fMRI demonstrated a relatively higher value in binary networks, consistent with group comparison results (Figure 3).

## 4 Discussion

### 4.1 Reduced small-world property and network efficiency

In our study, both RE patients and healthy controls exhibited consistent modifications in the network connectivity characteristics across both binary and weighted networks as the sparsity threshold varied, suggesting that the brain networks of RE patients maintained normal structural and functional properties. Notably, the brain networks of RE patients exhibited the characteristic small-world architecture, with γ > 1 and λ ≈ 1. This persistence of small-world property suggests that information transfer within the brain during cognitive or motor activities continues to be facilitated in RE patients, consistent with a previous study (Song et al., 2021).

However, significant differences are observed in the small-world property between RE patients and healthy controls. In our findings, both rs-fMRI and es-fMRI exhibited lower σ, Cp, and γ and higher Lp values in brain networks compared to healthy controls. This implies that RE patients possess a weaker small-world network than their healthy controls. The small-world property measures the equilibrium between global and local processing mechanisms (Guan et al., 2022). Elevated Cp and γ values signify functional segregation within brain networks, indicating a prevalence of localized interconnectivity (Guan et al., 2022). Conversely, reduced Lp and λ values may suggest functional integration within the brain, reflecting the ability to transmit global information (Guan et al., 2022). In our study, the decreased Cp and γ could suggest a weakening of the local brain networks, leading to decreased interconnections among neighboring brain regions and impeding effective communication. From a network topology perspective, this might result in diminished or severed functional connectivity between certain brain regions, effectively excluding them from processing pertinent brain activity. Simultaneously, the increased Lp could indicate a reduction in the communication efficiency of global networks, thereby inhibiting the comprehensive transmission of information during normal brain activity. This conclusion is further reinforced by the poorer network efficiency parameters (Eg and Eloc) in RE patients compared to healthy controls.

Whereas the findings of small-world property have not been consistent in previous similar studies. Consistent with our results, Jiang et al. (2017) found that the functional networks of right TLE tended to have more random attributes with reduced σ. During the interictal period, the neural network moved into a more randomly organized state with higher Lp and decreased Cp (Ponten et al., 2007). However, Wang J. et al. (2014) discovered that TLE patients exhibited statistically significant increases in Cp and Lp in comparison to the controls. Sethi et al. (2016) analyzed task-free fMRI data in polymicrogyria patients and found higher Cp and Lp in the affected area relative to the contralateral regions, indicating that the lesional anomalies may contribute disproportionately to global modifications. The reasons for the inconsistent findings remain unclear. They may be attributed to differences in sample sizes, patients’ ages and epilepsy phenotype, methods of measuring the connection form, use of ASMs as well as different experimental techniques (Jiang et al., 2017). Network properties appear to correlate with epilepsy phenotypes, and brain network organization seem to be modulated by the specific lesional and histopathological subtype (Tavakol et al., 2019). The RE patients we studied were in a resting state, and we believe that the course of the disease and the state of the disease could also be the factors that influenced the results of the study. In summary, previous studies and our findings indicate elevated Lp with concomitant increases or decreases in Cp within the brain networks of patients with TLE or RE. This suggests that the efficiency of information transfer and integration within the brain networks of these individuals with epilepsy was impaired.

Our study demonstrated the potential decline in the small-world architecture of brain networks among RE patients, indicated by decreased Cp, σ and γ and increased Lp. The brain networks of RE patients may lean toward a more random network structure. Such a shift toward randomness could compromise the stability and coordination of network connectivity between different brain regions, leading to a potential lack of synchronized and systematic responses to external stimuli or spontaneous brain activity. This observation implies that certain brain functions may be compromised or impaired in RE patients. Similar studies have also reported the relationship between small-world parameters and brain dysfunction. For example, Wang X. B. et al. (2014) found that the γ and λ were increased in the patients with mild cognitive impairment, and these abnormalities were associated with the slow speed of information processing in brain networks. Bai et al. (2012) and Wang et al. (2013) demonstrated a positive correlation between higher Lp values and poorer cognitive performance, as evidenced by clinical manifestations such as emotional, cognitive, or language impairment. The authors (Hatlestad-Hall et al., 2021) investigated the correlation between σ and functional neurocognitive networks in focal epilepsy and found that the σ of the default mode network (DMN) was associated with memory performance in patients.

### 4.2 Decreased activity in certain brain regions affects relevant brain functions

Additionally, RE patients exhibited significant disparities in nodal attributes compared to healthy controls. Bc measures the ability of a node to efficiently transmit information in networks by assessing its contribution to the shortest path between all other pairs of points (Guan et al., 2022). Dc represents the sum of direct connections of a node in a network (Guan et al., 2022). An elevated Dc indicates more connections (Rubinov and Sporns, 2010), implying a central hub status for the node. Variations in Bc and Dc within relevant brain regions indicate differences in transmission efficiency and connectivity strength. Notably, differences in Bc exhibited weakened transmission efficiency in the thalamus and fusiform gyrus among RE patients in our study. The thalamus is a relay station that receives sensory inputs from the ascending reticular activating system and transmits them to cortical areas (Sun et al., 2021), thereby maintaining heightened alertness and vigilance across the brain (Sun et al., 2021). The fusiform gyrus, located in the visual association cortex of the temporal lobe, is mainly responsible for face recognition. This result implies a potential impairment in information transmission ability and visual function among RE patients, affecting normal processing efficiency. Conversely, RE patients exhibited elevated Bc values in the right putamen, indicating the retention of motor control and postural coordination abilities. Additionally, an elevated Dc in the precuneus and supramarginal gyrus signifies increased connections with other brain areas, suggesting that the parietal lobe can serve as a highly connected hub in the brain networks of RE patients. By contrast, decreased Dc values in ROL.L, pallidum, and thalamus suggest reduced activity or connections within and around these brain areas. Notably, the precuneus, a part of the DMN, is activated during periods of rest and relatively inactivated during external stimulus tasks, thereby maintaining the equilibrium of brain networks (Raichle et al., 2001; Luo et al., 2011). Its activation enables the continuous acquisition of information from the environment and the body (Raichle et al., 2001). Meanwhile, the supramarginal gyrus plays a role in complex movements. The enhanced connectivity observed in the precuneus and supramarginal gyrus might compensate for impaired efficiency of information, allowing basic information processing and activity capability maintenance.

NCp serves as an indicator of a local network connectivity strength or capacity of a node. Notably, RE patients exhibited increased NCp in the precentral gyrus and relatively diminished NCp in regions, such as the parahippocampus, occipital lobe, putamen, and temporal lobe, indicating that the local connectivity strength of brain networks in RE patients is lower compared to healthy controls. The precentral gyrus, located in the frontal lobe, is associated with somatomotor functions. The frontal lobe plays a crucial role in various higher-order cognitive functions, behavioral control, and somatomotor and somatosensory functions, which are actively engaged in the sustained consciousness of epilepsy (Caplan et al., 2008). This emphasizes the significance of the frontal lobe in information processing in the brain networks among RE patients. The parahippocampus, forming a part of the hippocampal circuit, is involved in higher neural functions, such as emotion, learning, and memory. The occipital lobe, primarily responsible for visual processing, is intricately connected to cognitive and behavioral control pathways, particularly about visuospatial ability. The superior temporal gyrus plays a vital role in sound processing. These observed findings suggest that the brain functions related to memory, learning, emotion, visuospatial, and sound may be impaired among RE patients. Compared to healthy controls, the discrepancy of NCp in INS.L between rs-fMRI and es-fMRI, suggesting the possible impact of electrical stimulation.

Our study demonstrates that both types of networks yield similar outcomes, indicating that the characteristics of network topological properties are prevalent in the brain networks of RE patients. Conducting a comprehensive analysis of both network types enhances the credibility of our findings. In our study, the results obtained from analyzing global and nodal network properties in RE patients are consistent and mutually reinforcing. The observed disruption of the small-world structure indicates a decline in local connections between different brain network regions, leading to a corresponding decrease in network transmission efficiency. Additionally, the nodal network properties exhibited decreased activity in the brain regions, such as the thalamus, temporal lobe, occipital lobe, basal ganglia, and parahippocampus, whereas increased activity in the frontal lobe and precuneus among RE patients compared to healthy controls. This suggests disrupted connectivity within the brain networks of RE patients, potentially impairing the efficiency of information transmission and network functionality. Notably, these alterations impact brain functions related to consciousness, movement, somatosensory, visual, and auditory processes, emphasizing the mechanisms underlying the observed abnormal brain functions in RE patients.

### 4.3 Reduced network connectivity among intra-modules and inter-modules

Compared to healthy controls, network connectivity patterns in RE patients exhibited relatively diminished connections and weakened connectivity strength across the entire brain (Figures 5A,B). This observation implies potential reduced activity and impaired functions within areas, such as the occipital network areas and subcortical areas. The reduced connections between the frontal lobe and subcortical areas and the nodal property results suggest a potential weakening or disruption in synchronized activity between the cortex and the subcortical areas (particularly the thalamus), in RE patients. A previous animal study suggested that the cortex and thalamus are oscillatory structures responsible for generating sleep spindles (Steriade et al., 1985). Thus, our results indicate that reduced cortical-thalamic connectivity may affect normal brain activity. The es-fMRI brain networks exhibited a modular feature, with enhanced connections in the brain regions, such as the parieto-occipital lobe (Figure 5A). Nodal properties showed that the Dc values of parietal lobe were higher in RE patients than those in healthy subjects, supporting this finding. Enhanced parieto-occipital connectivity may be a remedy to offset the general reduced connections within inter-modules and intra-modules, allowing RE patients to maintain the basic functions of brain networks. These alterations in network connectivity among RE patients, even during resting-state, could serve as a compensatory mechanism before structural and functional damage ensues. This theory emphasizes the significance of functional connectivity changes before structural changes in certain conditions (Xu et al., 2019; Guan et al., 2022). The increased modular connectivity strength in the es-fMRI brain networks following electrical stimulation can be attributed to the activation of activity between specific brain regions through electrical stimulation, resulting in enhanced connectivity.

### 4.4 Electrical stimulation can regulate brain activity through network systems

Compared to rs-fMRI, es-fMRI showed comparatively lower σ and γ, as well as increased Lp in two network types and Cp in binary networks. The comparison of network property results among three groups offers additional support for these results. In general, the brain networks of es-fMRI displayed a weaker small-world property and a lower overall efficiency of information transmission. This increased local network connectivity (increased Cp) can be attributed to the effect of electrical stimulation, which causes certain regions of the brain to become more active. It indicates that electrical stimulation may play a significant role in influencing or modulating network activity or connectivity within the brain. Nodal properties suggest that electrical stimulation can contribute to the observed increases or decreases in the activity of certain brain regions compared to their pre-electrode implantation states. This modulation of brain network activity subsequently leads to changes in connectivity patterns, thereby affecting network properties, such as the small-world property.

Our present findings suggest that electrical stimulation may result in less efficient neuronal networks (a weaken small-world network), leading to decreased synchronized connectivity throughout the brain. We attribute the reasons for this outcome to several factors. First, there is a slight signal attenuation resulting from electrode implantation (Thompson et al., 2020). Though the impact is negligible after undergoing quality control and data preprocessing, it cannot be disregarded entirely. Second, various stimulation sites could produce distinctive effects on the characteristics of brain network, because each targeted stimulus might have a different function in brain network pathways. For example, the amygdala is a part of the limbic system and is associated with emotions. The frontal lobe is responsible for many higher cognitive functions, such as language and motor functions. Therefore, their regulation of brain activity might be different, which can lead to different network phenotypic characteristics. Based on the data conditions of our study, the effect of electrical stimulation with the amygdala as the main stimulus target may produce lower network efficiency results (Supplementary Table 1). Of course, more researches are needed to support this hypothesis, which will be improved in future study. Third, electrical stimulation does not solely activate the brain activity surrounding the stimulus point, but produces particular effects by regulating the activity of brain regions through a network system (Fox et al., 2014; Thompson et al., 2020; Vetkas et al., 2022b). Stimulation delivered through implanted electrodes can trigger activation in specific brain regions and enhance activity. By contrast, other regions of the brain may exhibit decreased activity or remain relatively inactive. Consequently, the interconnectivity between brain regions may undergo modification, thereby regulating the flow of information within the original network pathway. It is possible that brain networks as a whole may exhibit a reduction in network efficiency even if enhanced activity in certain brain regions. Combined with the increased Cp, nodal attributes and modular results of es-fMRI, we support the possibility of the latter two hypotheses.

However, the usefulness of this regulatory effect (activation or inhibition) for disease-specific brain networks varies in different circumstances. In other words, whether the electrical stimulation can achieve the desired therapeutic effect is dependent on the pathogenesis, severity of lesions (Fox et al., 2014), and network mechanism of particular diseases. RE, for instance, is primarily caused by recurrent and uncontrollable abnormal synchronous discharges from brain neurons. The treatment approach is to inhibit or terminate these discharges instead of activating them. If the stimulation in a particular area of the brain can regulate the interconnectivity of regions along the neural network pathway, resulting in reduced connectivity strength and efficiency, and ultimately inhibit aberrant synchronous neuronal activity, then this could advance the research and clinical implementation of electrical stimulation for the treatment of RE. Therefore, there is a need for further experimental research to investigate the regulatory effects of electrical stimulation on the brain network systems in the treatment of specific diseases like RE. Several important issues need to be addressed, such as determining the optimal placement of implanted electrodes and effectively managing the regulatory effects. Various brain targets have been explored for deep brain stimulation (DBS) in RE. Targets that have been investigated in randomized controlled trials and are currently used clinically include the anterior nucleus of the thalamus (ANT), the centromedian nucleus of the thalamus (CMT), and the hippocampus (Zangiabadi et al., 2019). The impact of identical target stimulation on distinct epilepsy phenotypes varies. A systematic review analysis suggests more efficient DBS of ANT for focal seizures, wider use of CMT for generalized seizures, and hippocampal DBS for temporal lobe seizures (Vetkas et al., 2022a). In our study, the electrical stimulation sites were predominantly situated in the amygdala and heschls gyrus. However, given the limited findings currently available, it would be premature to conclude if the electrical stimulation at these targets can lead to more effective therapeutic outcomes. To achieve therapeutic benefits, it is advisable to target brain regions that have the potential to induce significant modulation of brain activity while simultaneously inhibiting or disrupting disease-related activities or connections. Future research will identify specific network connectivity models and corresponding network attributes from diverse stimulation sites of individuals with RE. Experimental verification is essential to investigate the changes in brain activity resulting from electrical stimulation. We propose that this study represents an essential initial step toward the comprehensive exploration of network alterations at the individual level following intracranial brain stimulation, which could potentially enhance clinical decision-making in the context of refractory neurological disorders.

## 5 Limitations

Our study had several limitations. First, it should be recognized that changes to the MRI scanner and parameters were introduced in the laboratory settings before and after electrode implantation, potentially influencing certain fMRI data. However, the authors of raw data conducted quality assessment and made a comparison with a larger sample of healthy individuals, to address this concern. Although the resulting es-fMRI data exhibited some noise and signal loss, these effects did not significantly impact the overall findings. As recommended by the authors (Thompson et al., 2020), data processing was primarily controlled to mitigate motion artifacts and noise, leading to the exclusion of relevant subjects and data. Second, the study faced limitations in establishing a correlation between patients’ clinical characteristics and network connectivity due to constraints in available data. Third, due to relatively small sample size and unbalanced scanning conditions and parameters of this dataset, it is not sufficient for us to draw firm conclusions about the causal effects of electrical stimulation. However, we used two types of networks in the study to improve the scientific quality of the results. And the results of global attributes, nodal attributes and modular analysis supported and complemented each other, which increased the credibility and repeatability of the results of this study. To further substantiate the effects of electrical stimulation, we performed a comparative analysis of the global network properties of the different stimulation points (Supplementary Table 1; Figure 1). Due to the small sample size of the data, only the global network properties in amygdala and heschls gyrus were compared, and there are currently no obvious differences other than network efficiency. In conclusion, our study still provides a lot of information to support the study for the characteristics of brain networks and possible effects of electrical stimulation in patients with RE.

## 6 Conclusion

Our findings emphasize a reduction in small-world property among RE patients, indicating a shift toward random networks. This alteration in network architecture, characterized by reduced connectivity between network regions, potentially undermines network stability and information transmission efficiency. The revised inter-brain network connectivity pattern observed in RE patients may implicate cognitive and behavioral regulation. Electrical stimulation is a promising avenue for regulating specific brain regions or broader network systems. This approach offers a valuable means to investigate the effects of electrical stimulation on brain network connectivity and may supplement existing methodologies. This provides a foundation for studying the mechanisms of brain networks in RE and developing interventions. In future studies, larger sample sizes and monitoring dynamic changes in functional images following electrode implantation in RE patients will significantly enhance research in this field.

## Data availability statement

Publicly available datasets were analyzed in this study. This data can be found at: https://openneuro.org/datasets/ds002799/versions/1.0.4.

## Ethics statement

The studies involving humans were approved by The University of Iowa Institutional Review Board, Stanford University, and Caltech. The studies were conducted in accordance with the local legislation and institutional requirements. Written informed consent for participation in this study was provided by the participants’ legal guardians/next of kin.

## Author contributions

YS: Formal analysis, Funding acquisition, Investigation, Methodology, Project administration, Supervision, Writing – original draft, Writing – review & editing. QS: Writing – original draft, Writing – review & editing. MY: Supervision, Writing – review & editing. AM: Project administration, Supervision, Writing – review & editing, Formal analysis, Investigation, Methodology.

## Figures and Tables

**Table:** 

RE	Refractory epilepsy
fMRI	Functional magnetic resonance imaging
rs-fMRI	The resting-state fMRI of RE patients
es-fMRI	The fMRI concurrent electrical stimulation of RE patients
HC-fMRI	Healthy controls’ fMRI
GRETNA	A graph theoretical network analysis toolbox for imaging connectomics
ASMs	Anti-seizure medications
CAE	Childhood absence epilepsy
TLE	Temporal lobe epilepsy
EPI	Echo-planar imaging
TR	Repetition time
TE	Echo time
TI	Inversion time
AUC	Area under the curve
FD	Framewise displacement
DMN	Default mode network
Sigma (σ)	Small-worldness
Cp	Clustering coefficient
Lp	Characteristic path length
Gamma (γ)	Normalized clustering coefficient`
Lambda (λ)	Normalized characteristic path length
Eg	Global network efficiency
Eloc	Local network efficiency
NCp	Nodal clustering coefficient
NLp	Nodal characteristic shortest path length
Bc	Betweenness centrality
Dc	Degree centrality
AUC	Area under the curve
FFG.L	Left fusiform gyrus
PUT.L	Left putamen
PUT.R	Right putamen
THA.L	Left thalamus
THA.R	Right thalamus
PAL.L	Left pallidum
PAL.R	Right pallidum
PCUN.R	Right precuneus
ROL.L	Left rolandic operculum
SMG.L	Left supramarginal gyrus
SOG. R	Right superior occipital gyrus
INS. L	Left insula
PHG. L	Left parahippocampal gyrus
PreCG. L	Left precental gyrus
STG. L	Left superior temporal gyrus
DBS	Deep brain stimulation
ANT	Anterior thalamic nucleus
CMT	Centromedian thalamic nucleus

## Footnotes

^1^ https://openneuro.org/datasets/ds002799/versions/1.0.4

^2^ http://fcon_1000.projects.nitrc.org/fcpClassic/FcpTable.html

^3^ http://www.nitrc.org/projects/gretna/

^4^ http://www.nitrc.org/projects/bnv/
