# Supplementary figures and images for: Topological properties and connectivity patterns in brain networks of patients with refractory epilepsy combined with intracranial electrical stimulation
Source: Front Neurosci. 2023 Nov 23;17:1282232. doi: 10.3389/fnins.2023.1282232 (PMC10701286; doi:10.3389/fnins.2023.1282232)

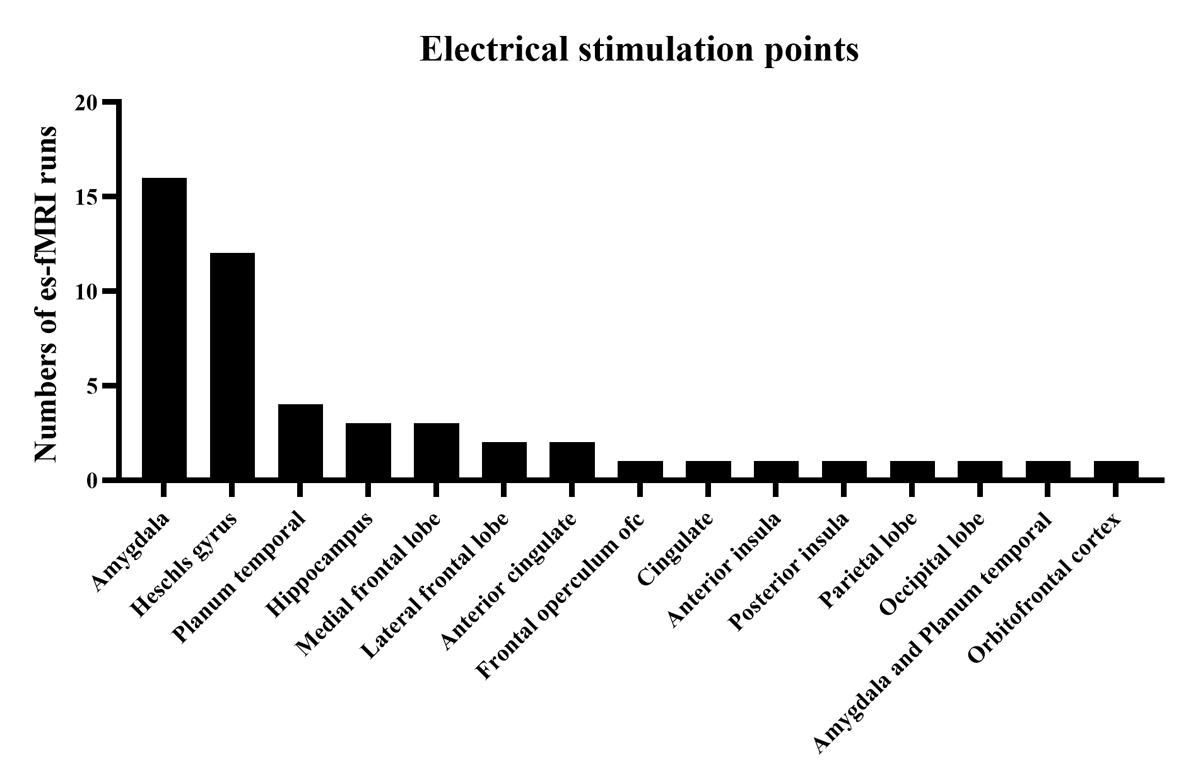

Supplement: Supplementary file 1 [file Image_1.tif]
