# Supplementary material for: Topological properties and connectivity patterns in brain networks of patients with refractory epilepsy combined with intracranial electrical stimulation
Source: Front Neurosci. 2023 Nov 23;17:1282232. doi: 10.3389/fnins.2023.1282232 (PMC10701286; doi:10.3389/fnins.2023.1282232)
Supplement: Supplementary file 2 [file Table_1.docx]

Supplementary Table 1 Comparisons of global network properties between different stimulation sites.

|  | Binary networks | | | Weighted networks | | |
| --- | --- | --- | --- | --- | --- | --- |
|  | Amygdala | Heschls gyrus | P -value | Amygdala | Heschls gyrus | P -value |
| aSigma | 0.49 ± 0.08 | 0.54 ± 0.08 | 0.196 | 0.46 ± 0.08 | 0.50 ± 0.07 | 0.161 |
| aCp | 0.17 ± 0.01 | 0.17 ± 0.01 | 0.162 | 0.07 ± 0.01 | 0.08 ± 0.01 | 0.111 |
| aLp | 0.69 ± 0.04 | 0.67 ± 0.04 | 0.212 | 1.00 ± 0.21 | 0.91 ± 0.16 | 0.205 |
| aGamma | 0.55 ± 0.10 | 0.62 ± 0.09 | 0.061 | 0.57 ± 0.11 | 0.64 ± 0.11 | 0.099 |
| aLambda | 0.33 ± 0.01 | 0.34 ± 0.01 | 0.053 | 0.37 ± 0.02 | 0.38 ± 0.03 | 0.232 |
| aEg | 0.14 ± 0.01 | 0.15 ± 0.01 | 0.024^*^ | 0.10 ± 0.02 | 0.11 ± 0.02 | 0.157 |
| aEloc | 0.21 ± 0.01 | 0.22 ± 0.05 | 0.016^*^ | 0.16 ± 0.03 | 0.17 ± 0.02 | 0.118 |

The table shows the AUC values comparison of the global network properties for stimulus points between the amygdala and the heschls gyrus in two types of networks. Data are represented as Mean ± SD. **p* < 0.05. The p-values are obtained using an independent samples t-test. The “a” refers to the AUC values of these network properties.

AUC: area under the curve; Sigma (σ): small-worldness; Cp: clustering coefficient; Lp: characteristic path length; Gamma (γ): normalized clustering coefficient; Lambda (λ): normalized characteristic path length; Eg: global network efficiency; Eloc: local network efficiency.
